# P-1349. Relative Impact of Antibiotic-Resistant vs Susceptible Infections in US Hospitals, 2018-2022: Comparing Mortality Rates and Absolute Death Counts

**DOI:** 10.1093/ofid/ofaf695.1537

**Published:** 2026-01-11

**Authors:** Alexander Lawandi, Christina Yek, Morgan Walker, Shanshan Liu, Maniraj Neupane, Guoqing Diao, Claire N Shappell, John P Dekker, Chanu Rhee, Michael Klompas, John H Powers, Sarah Warner, Sameer S Kadri

**Affiliations:** 1. Critical Care Medicine Department, Clinical Center, National Institutes of Health, Bethesda, MD, Montreal, Quebec, Canada; National Institute of Allergy and Infectious Diseases, Bethesda, Maryland; National Institutes of Health, Bethesda, Maryland; The George Washington University, Washington, District of Columbia; 1. Critical Care Medicine Department, Clinical Center, National Institutes of Health, Bethesda, MD, 2. Critical Care Medicine Branch, National Heart Lung and Blood Institute, Bethesda, MD, Bethesda, Maryland; George Washington University, Washington, District of Columbia; Brigham and Women's Hospital, Boston, Massachusetts; National Institute of Allergy and Infectious Diseases, Bethesda, Maryland; Brigham and Women's Hospital / Harvard Medical School, Boston, MA; Harvard Medical School and Harvard Pilgrim Health Care Institute, Boston, Massachusetts; Support to National Institute of Allergy and Infectious Disease, Bethesda, MD; NIH - Critical Care Medicine Department, Bethesda, MD; National Institutes of Health Clinical Center, Bethesda, MD

## Abstract

**Background:**

Clinical guidance, research, and burden estimates prioritize antibacterial resistant organisms. However, understanding the relative burden of deaths due to resistant vs susceptible organisms might tailor clinical and public health priorities. We therefore contrasted mortality rates against absolute death counts associated with resistant vs susceptible infections in U.S. hospitals.

**Figure 1:**
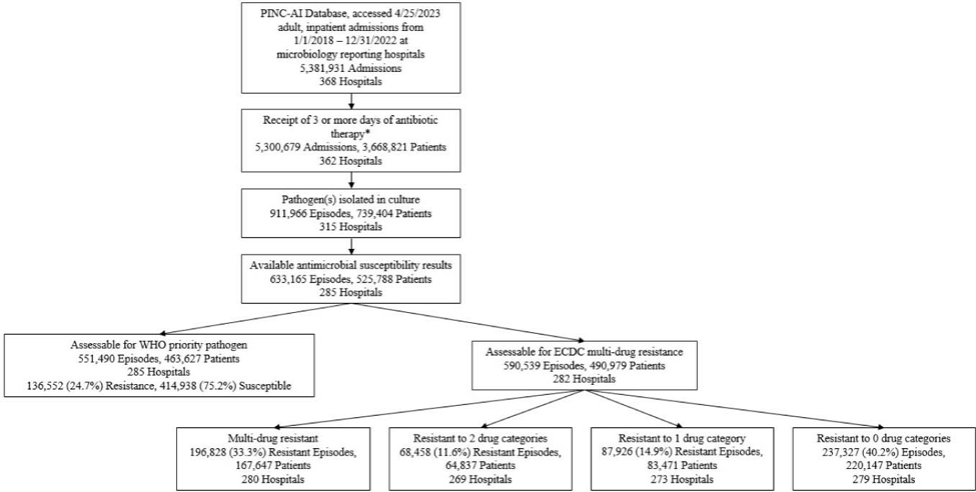
Cohort Selection Flowsheet. The PINC-AI database was queried to identify adult inpatients receiving 3 or more days of antibiotics in conjunction with the isolation of a bacterial pathogen from culture. WHO and ECDC resistance definitions were applied to determine the prevalence and mortality rates of resistant and susceptible pathogens of interest.* Exceptions were made for inclusions of death after 2 days of antibiotic therapy

**Figure 2:**
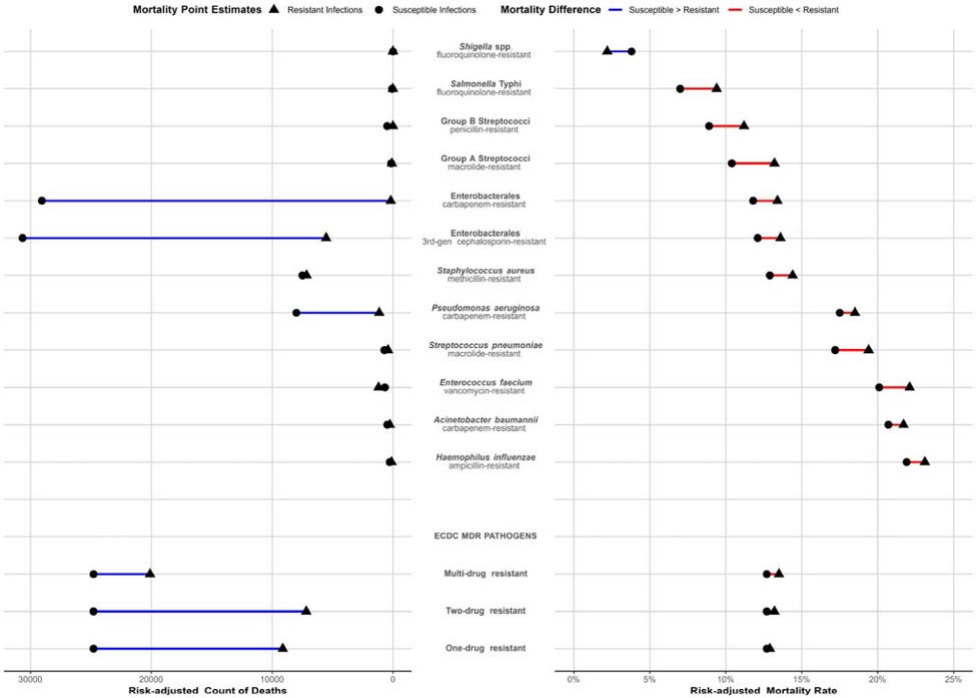
Resistant versus susceptible organism-specific risk-adjusted mortality count and risk-adjusted mortality rate for WHO Priority Pathogen and ECDC MDR pathogen defining lenses. For WHO lens, “susceptible” refers to tracer antibiotic (e.g., fluoroquinolone susceptible Shigella spp.) . For ECDC lens, “susceptible” refers to all the antibiotic categories in the ECDC MDR defining rubric [Magiorakos, Clin Micro Infect 2012]. Mortality models were adjusted for patient demographics, comorbidity burden, admit year, admit source, POA Do not resuscitate status, infection onset, polymicrobial status, and individual POA acute organ failure score elements [Courtright. Crit Care Med 2017]. Note: S. pneumonia counts do not include antigen-based identification. diagnostics not included. POA= present-on-admission

**Methods:**

In this retrospective cohort study, adult inpatients with bacterial infection (culture growth and receiving antibiotics for > 3 days) were identified in the PINC-AI database. Resistance was defined by 2024 WHO Priority Pathogen and ECDC multidrug resistance (MDR) lenses. Predictive margins were used to obtain risk-adjusted mortality rates while controlling for confounders. To compare infection- vs resistance-related mortality, adjusted odds ratio (aOR) for mortality in matched patients hospitalized for similar conditions with (vs without) hospital-onset infection were compared.

**Figure 3:**
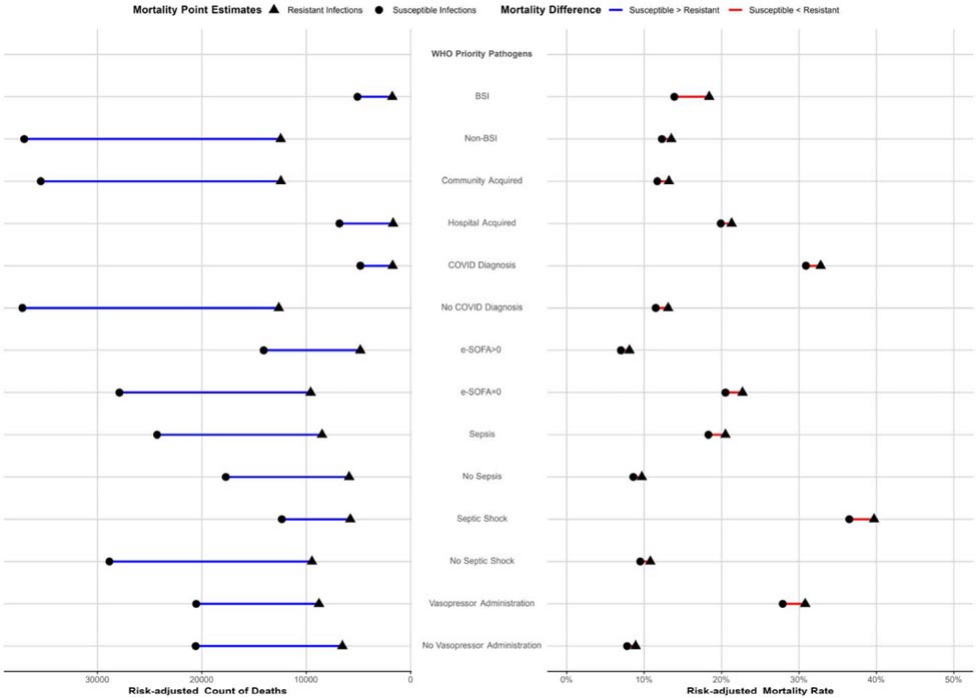
Subgroup Analysis of Resistant versus susceptible organism specific risk-adjusted mortality counts and risk-adjusted mortality rates for WHO Priority Pathogen defining lens.

For WHO priority pathogen lens, “susceptible” refers to susceptible to the tracer antibiotic. Sepsis, septic shock and COVID-19 defined using explicit ICD-10 coding. E-SOFA= Electronic Sequential Acute Organ Failure Score. BSI=bloodstream infection

**Table 1:**
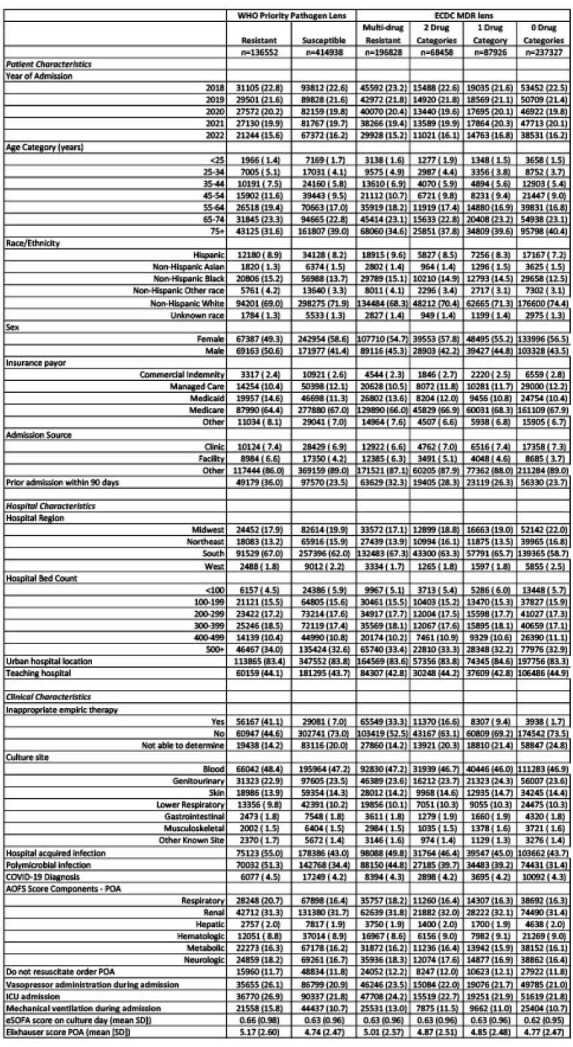
Demographic table stratified by resistance definition and status.

**Results:**

At 285 hospitals between 2018-2022, 739,404 inpatients had 911,966 bacterial infections (Fig.1) of whom 10.7% died. Patients with infections due to resistant (vs susceptible) pathogens had more critical illness and inadequate empiric therapy (Table). Risk-adjusted mortality was marginally higher for resistant (vs susceptible) infections (11.1 vs 10.1% for WHO priority pathogens and 10.0% vs 9.6% vs 9.7% vs 9.4% for resistance to 3+(MDR), 2, 1 and 0 antibiotic categories, respectively; Fig.2). Modeled absolute deaths across all pathogens (except E. faecium) and subgroups were higher for susceptible infections (Figures 2 and 3). Among patients hospitalized for other reasons, hospital-onset infection itself was associated with greater excess odds of mortality than resistance (aOR resistant vs no infection= 1.55(95% CI. 1.48-1.63); aOR susceptible vs no infection=1.41(95% CI. 1.37-1.45).

**Conclusion:**

In US hospitals, risk-adjusted mortality rates are slightly higher for patients with resistant vs susceptible infections, but susceptible infections are more common and thus account for 2-3-fold more deaths compared to resistant organisms. Both antibiotic susceptible and resistant bacterial infections should be prioritized for prevention, management and research.

**Disclosures:**

All Authors: No reported disclosures

